# The Impact of Two Cordless Retraction Materials on Inflammatory Biomarkers Interleukin-1 Beta and Tumor Necrosis Factor-Alpha in Gingival Crevicular Fluid: An In-Vivo Study

**DOI:** 10.7759/cureus.89464

**Published:** 2025-08-06

**Authors:** Sugandhi Pidaparthi, Kadiyala Krishna Kishore, Haragopal Surapaneni, Naga Phanindra Madhavarapu, Nayeema Sultana, Sowrabha Adavikatla, Seema Gupta

**Affiliations:** 1 Department of Prosthodontics, Sibar Institute of Dental Sciences, Guntur, IND; 2 Department of Orthodontics, Kothiwal Dental College and Research Centre, Moradabad, IND

**Keywords:** biomarker, gingival crevicular fluid, gingival retraction techniques, interleukin-1, tumor necrosis factor-alpha

## Abstract

Introduction: This study aimed to evaluate and compare the inflammatory responses of two cordless gingival retraction systems by assessing the levels of interleukin-1 beta (IL-1β) and tumor necrosis factor-alpha (TNF-α) in gingival crevicular fluid (GCF). The objectives were to measure IL-1β and TNF-α levels before and after retraction using kaolin-based and polyvinyl acetate-based cordless gingival retraction materials at baseline, 1 h, and 24 h, and to compare the inflammatory profiles of these materials.

Materials and Methods: This in-vivo comparative study used a split-mouth design, with each of the 10 enrolled patients serving as their own control by receiving both kaolin-based paste (Traxodent®, Premier Dental Products, Plymouth Meeting, PA, USA) and polyvinyl acetate-based strip (Merocel®, Medtronic, Minneapolis, MN, USA) on different endodontically treated teeth requiring full-coverage crowns, thereby minimizing interindividual variability. The allocation of both agents to specific teeth was systematically determined based on clinical suitability and tooth position, alternating between the contralateral teeth to ensure consistent intraoral conditions. After oral prophylaxis and a 7-day washout period, baseline GCF samples were collected using sterile absorbent paper point (Dentsply Sirona, Charlotte, NC, USA). Traxodent®, a kaolin-based material, was applied to one tooth, and Merocel®, a polyvinyl acetate-based material, was applied to another tooth in each patient. GCF samples were collected at baseline, 1 and 24 h post-retraction, stored at -70^0^C, and analyzed for IL-1β and TNF-α using enzyme-linked immunosorbent assay (ELISA) kits (Elabscience, New Delhi, India). Data were analyzed using independent t-tests for intergroup comparisons and repeated measures analysis of variance (ANOVA) with Bonferroni post-hoc tests for intragroup comparisons (p < 0.05).

Results: Both materials significantly increased IL-1β and TNF-α levels after 1 h. The polyvinyl acetate-based strip showed sustained IL-1β elevation at 24 h, whereas the IL-1β levels of the kaolin-based paste declined. Conversely, kaolin-based paste maintained elevated TNF-α levels at 24 h, whereas the polyvinyl acetate-based strip’s TNF-α level decreased significantly.

Conclusion: Both cordless retraction systems induced acute inflammation, with polyvinyl acetate-based strips causing prolonged IL-1β elevation and kaolin-based paste sustaining TNF-α levels. These findings suggest material-specific inflammatory responses, which can guide clinicians in selecting retraction systems to balance efficacy and periodontal health.

## Introduction

Management of gingival tissues is a critical aspect of restorative dentistry, particularly when fabricating fixed dental restorations with finish lines at or near the gingival sulcus [1]. This anatomical groove, which separates the tooth from the surrounding periodontal tissues, often requires careful manipulation to ensure accurate and precise impression-making. Gingival retraction is a pivotal step in this process, facilitating a clean, dry, and accessible working field and allowing impression materials to effectively capture subgingival details. Traditional gingival retraction methods include mechanical, chemical, surgical, and combined approaches [2,3]. Mechanical retraction typically involves the use of retraction cords that physically displace the gingival tissues, whereas chemical methods employ hemostatic agents to induce vasoconstriction and tissue shrinkage [1]. Surgical techniques, such as gingivectomy, are more invasive and can directly modify soft tissues [4]. However, prolonged placement of retraction cords or the use of potent chemical agents has been associated with increased gingival trauma and inflammation, potentially compromising periodontal health [5].

In response to these challenges, cordless retraction systems have emerged as innovative alternatives to enhance clinical efficiency and patient comfort [1]. These systems eliminate the need for traditional retraction cords and offer a less invasive approach to gingival displacement [6]. By minimizing tissue trauma and reducing procedural complexity, cordless retraction materials are increasingly being adopted for procedures such as crown impressions and restorative treatments [6,7]. Despite their growing popularity, the biological impact of these materials on periodontal tissues, particularly inflammatory responses, remains unexplored. Understanding these effects is essential to ensure that such innovations do not adversely affect the long-term health of the periodontium. Martins et al. conducted a systematic review to compare the efficacy of conventional cord and cordless systems for gingival displacement [7]. They included nine studies and concluded that the cord technique resulted in increased displacement compared with the cordless technique.

Gingival crevicular fluid (GCF), a serum-like exudate found within the gingival sulcus, is a valuable medium for assessing periodontal health. GCF contains various biomolecules, including inflammatory biomarkers such as interleukin-1 beta (IL-1β) and tumor necrosis factor-alpha (TNF-α), which are critical mediators of immune responses in periodontal tissues [8]. Elevated levels of IL-1β and TNF-α in the GCF are indicative of local inflammatory activity, often triggered by dental procedures or materials [9]. Monitoring these biomarkers provides insights into the biocompatibility of retraction materials and their potential to induce or exacerbate gingival inflammation. As cordless retraction systems become more integrated into clinical practice, evaluating their effects on IL-1β and TNF-α levels in GCF is crucial for optimizing treatment outcomes and safeguarding periodontal integrity [10].

This study evaluated and compared the levels of IL-1β and TNF-α in the GCF before and after gingival retraction using two different cordless gingival retraction systems. The objectives of this study were to: evaluate the levels of IL-1β and TNF-α in GCF before and after gingival retraction using kaolin-based and polyvinyl acetate cordless retraction materials at baseline (0 h), 1 hour, and 24 h post-retraction, and to compare the effectiveness of kaolin-based and polyvinyl-based cordless retraction materials in modulating IL-1β and TNF-α levels in GCF at the same time points (0, 1, and 24 h) before and after retraction.

## Materials and methods

Study design and setting

This in-vivo study was conducted in the Department of Prosthodontics at the Sibar Institute of Dental Sciences, Guntur, India, from June 2023 to June 2024. A split-mouth design was employed, with each patient receiving both materials on different teeth, to minimize interindividual variability. This study was approved by the Institutional Ethical Committee of the Sibar Institute of Dental Sciences (Pr.187 /IEC/SIBAR/2023)and adhered to the principles of the Declaration of Helsinki. Written informed consent was obtained from all patients after thorough explanation of the study’s purpose, procedures, potential risks, and benefits, ensuring voluntary participation and ethical compliance.

Sample size estimation

The sample size was calculated using the G*Power software (version 3.1.9.2, Heinrich-Heine-Universität Düsseldorf, Düsseldorf, Germany). The parameters included 80% power, 5% alpha error, and an effect size of 0.3, derived from a reference study comparing TNF-α levels between two retraction cord groups across multiple time points [10]. Repeated measures within factors determined a minimum of 20 samples (GCF from two endodontically treated teeth per patient, incorporating two groups). Therefore, 10 patients were required in a split-mouth design, each providing two teeth needing full crowns, ensuring adequate power to detect significant differences.

Eligibility criteria

Patients were selected from patients attending the department, aged 20-40 years, requiring tooth preparation for full-coverage restorations, and exhibiting good gingival and periodontal health, as confirmed by clinical assessments. Exclusion criteria included patients with autoimmune diseases, systemic illnesses, periodontal diseases, those taking antibiotics or anti-inflammatory drugs, pregnant or lactating women, smokers, tobacco chewers, and alcohol users.

Methodology

**Figure 1:**
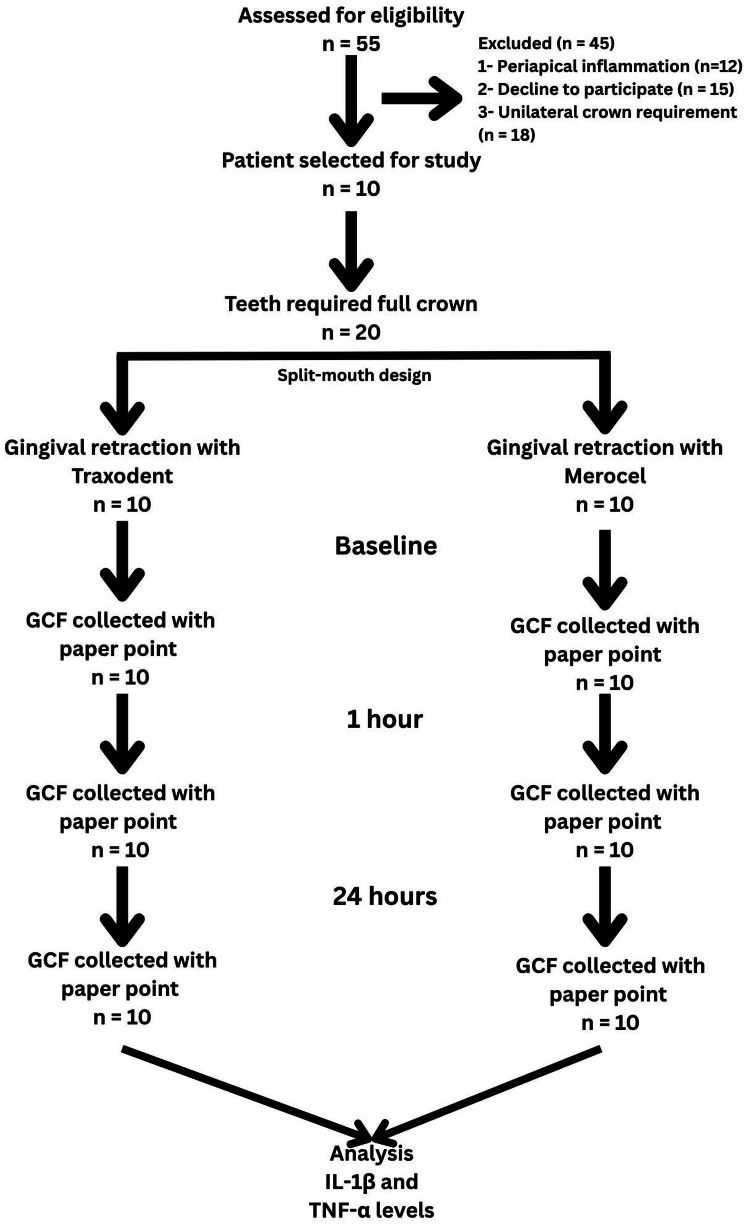
Study flow diagram Traxodent represents a kaolin-based cordless retraction paste, Merocel represents a polyvinyl acetate strip-based material. GCF: gingival crevicular fluid; IL-1β: interleukin-1 beta; TNF-α: tumor necrosis factor-alpha

Ten patients requiring full-coverage crowns for endodontically treated teeth (bilaterally) were enrolled in the study (Figure 1)

The selection adhered to strict inclusion and exclusion criteria to ensure the consistency of the results. To standardize oral hygiene, all patients underwent thorough oral prophylaxis, followed by a 7-day washout period to allow the gingival tissues to return to a healthy baseline state, thus minimizing the influence of recent cleaning on GCF composition. At the initial visit, periodontal health was evaluated using the Gingival Index to assess gingival inflammation and the Plaque Index to measure plaque accumulation, establishing a baseline for oral health prior to intervention [11].

Following the washout period and tooth preparation, baseline GCF samples were collected using sterile absorbent paper points (Dentsply Sirona, Charlotte, NC, USA, 06 taper) that were gently placed in the gingival sulcus of the selected teeth to absorb fluid without causing irritation or trauma. Each patient received both retraction materials in a split-mouth design: Traxodent® (Premier Dental Products, Plymouth Meeting, PA, USA), a kaolin-based cordless retraction paste, was injected into the sulcus of one tooth (n = 10) and Merocel® (Medtronic, Minneapolis, MN, USA), a polyvinyl acetate strip-based material, was gently packed into the sulcus of another tooth using Fischer’s Ultra Pak Packer (Ultradent Products, South Jordan, UT, USA) (n = 10).

One hour after retraction, GCF samples were collected from both teeth using sterile absorbent paper points to evaluate the immediate inflammatory response to the materials. A final set of GCF samples was collected 24 h post-retraction using absorbent paper points to monitor delayed or sustained inflammation induced by the materials. All GCF samples were labeled and stored in Eppendorf tubes (Eppendorf AG, Hamburg, Germany) and immediately deep-frozen at -700C to preserve the integrity of the inflammatory markers. The samples were thawed for 24 h before analysis.

Samples were diluted 1:100 with Sorensen’s buffer (0.05% bovine serum albumin in phosphate-buffered saline, pH 7.0), centrifuged at 2,000 rpm for 1 min, and stored at -700C until assayed. IL-1β levels were measured using a human IL-1β enzyme-linked immunosorbent assay (ELISA) kit (Elabscience, New Delhi, India), and TNF-α levels were measured using a Human TNF-α ELISA Kit (Elabscience, New Delhi, India). Optical density was measured at 450 nm using a Bio-Rad iMark Microplate Reader (Bio-Rad Laboratories, Hercules, CA, USA) at Zed Labs, Guntur, India.

Calibration and reliability

To ensure consistency, all clinical procedures, including GCF collection and retraction material application, were performed by a single trained operator to minimize procedural variability. The operator was calibrated through training sessions to standardize the placement of absorbent paper points and application of retraction materials. The Gingival Index and Plaque Index assessments were conducted by a single examiner, with intra-examiner reliability tested using duplicate measurements on a subset of patients, achieving a Cohen’s kappa coefficient of ≥ 0.85 for both indices. The ELISA were conducted in duplicate for each sample, and the microplate reader was calibrated before each use to ensure accurate optical density readings, with a coefficient of variation maintained below 10% for reliable biomarker quantification.

Statistical analysis

Data were analyzed using the Statistical Package for the Social Sciences (SPSS) version 20 software (IBM Corp., Armonk, NY, USA). The Shapiro-Wilk test was used to confirm the normal distribution of all variables, and the data were normally distributed. Data are expressed as the mean ± standard deviation. Intergroup comparisons of IL-1β and TNF-α levels in GCF between the kaolin-based paste (G1) and polyvinyl acetate-based strip (G2) groups were conducted using independent t-tests. Intragroup comparisons across time points (0h, 1h, 24h) were evaluated using repeated measures analysis of variance (ANOVA), followed by Bonferroni post-hoc tests to detect specific differences. Statistical significance was set at p < 0.05, ensuring reliable identification of differences in inflammatory marker levels between and within groups over time.

## Results

Table 1 presents the demographic details of study population. The study population comprised 6 (60%) males and 4 (40%) females, with mean ages of 38.5 ± 4.5 years and 41.5 ± 2.1 years, respectively. These demographic findings highlight the sex-balanced recruitment in the study to eliminate potential sex-based variations.

**Table 1:** Demographic details of study population. Number of patients are presented in frequency (n) and percentage (%), and age is represented as mean and standard deviation (SD).

Category	n (%)	Age in years (Mean ± SD)
Male	6 (60%)	38.5 ± 4.5
Female	4 (40%)	41.5 ± 2.1

The intergroup comparison of mean IL-1β levels in the GCF between the kaolin-based paste (G1) and polyvinyl acetate-based strip (G2) retraction cordless groups showed no significant difference at baseline (p = 0.937), indicating similar initial inflammatory profiles. At 1 h, G2 had higher IL-1β levels (245.3 pg/mL vs. 189.9 pg/mL), approaching significance (p = 0.081), suggesting greater early inflammation in G2. By 24 h, G2 exhibited significantly higher IL-1β levels (200.1 pg/mL vs. 160.1 pg/mL; p = 0.043), indicating sustained inflammation (Table 2).

**Table 2:** Intergroup comparison of mean interleukin-1 beta (IL-1β) levels in gingival crevicular fluid (GCF) in picograms/mL (pg/mL) at multiple time intervals. *p < 0.05 denotes statistical significance using independent t-test, G1 represent group using kaolin-based cordless retraction material, G2 represents group using polyvinyl acetate-based cordless retraction material. Data are presented as mean and standard deviation (SD), where n denotes number of patients in each group as a split-mouth study.

Time intervals	Groups	Mean	Median	SD	t value	p-value
0 h	G1 (n = 10)	136	146.5	29.37	0.08	0.937
	G2 (n = 10)	137.2	139.5	36.78		
1 h	G1 (n = 10)	189.9	182	49.77	1.85	0.081*
	G2 (n = 10)	245.3	222.5	74.43		
24 h	G1 (n = 10)	160.1	160	12.87	2.17	0.043*
	G2 (n = 10)	200.1	192	53.68		

The intergroup comparison of mean TNF-α levels in GCF between G1 and G2 cordless retraction groups revealed no significant differences at baseline (0 h; p = 0.9), indicating similar initial inflammatory profiles. At 1 h, G2 showed higher TNF-α levels (277.5 pg/mL vs. 248.8 pg/mL; p = 0.5), though not significant, suggesting a slightly greater early inflammatory response. At 24 h, G2 had higher TNF-α levels (250 pg/mL vs. 206.7 pg/mL; p = 0.36), but the differences were not significant. Both cordless retraction groups appeared to induce comparable gingival inflammation, with polyvinyl acetate-based strips potentially eliciting a slightly prolonged response (Table 3).

**Table 3:** Intergroup comparison of mean tumor necrosis factor-alpha (TNF-α) levels in gingival crevicular fluid (GCF) in picograms/mL (pg/mL) at multiple time intervals. p > 0.05 denotes no statistical significance using independent t-test, G1 represent group using kaolin-based cordless retraction material, G2 represents group using polyvinyl acetate-based cordless retraction material. Data are presented as mean and standard deviation (SD), where n denotes number of patients in each group as a split-mouth study.

Time	Groups	Mean	Median	SD	t value	p-value
0 h	G1 (n = 10)	191.2	177	70.48	0.13	0.9
	G2 (n = 10)	186.6	157.5	79.7		
1 h	G1 (n = 10)	248.8	211	87.54	0.68	0.5
	G2 (n = 10)	277.5	271	90.98		
24 h	G1 (n = 10)	206.7	170.5	86.71	0.93	0.36
	G2 (n = 10)	250	210.5	107.72		

The within-group comparison of IL-1β levels in the GCF for G1 and G2 cordless retraction groups showed significant temporal changes. In G1, IL-1β levels increased significantly from baseline (0 h: 136 ± 29.37 pg/mL) to 1 h (189.9 ± 52.46 pg/mL; p = 0.007), then decreased non-significantly by 24 h (160.1 ± 13.57 pg/mL; p = 0.425), indicating a transient inflammatory peak (p= 0.015). In G2, IL-1β levels rose significantly from baseline (137.2 ± 36.78 pg/mL) to 1 h (245.3 ± 78.46 pg/mL; p = 0.001) and remained elevated at 24 h (200.1 ± 56.59 pg/mL; p = 0.005), suggesting sustained inflammation (p = 0.003). Both materials induced acute gingival irritation at 1 h, with the polyvinyl acetate-based strip causing a stronger and more persistent inflammatory response (Table 4).

**Table 4:** Intragroup comparison of mean interleukin-1 beta (IL-1β) levels in gingival crevicular fluid (GCF) in picograms/mL (pg/mL) at multiple time intervals. *p < 0.05 denotes statistical significance using repeated analysis of variance with Bonferroni post-hoc test, G1 represent group using kaolin-based cordless retraction material, G2 represents group using polyvinyl acetate-based cordless retraction material. Data are presented as mean and standard deviation (SD), where n denotes number of patients in each group as a split-mouth study.

Groups	Time	Mean ± SD	F value	p-value	Repeated measure	Mean difference	t value	p-value
G1 (n = 10)	0 h	136 ± 29.37	8.46	0.015*	0h - 1h	-53.9	-3.39	0.007*
	1 h	189.9 ± 52.46			0h - 24h	-24.1	-1.51	0.425
	24 h	160.1 ± 13.57			1h - 24h	29.8	1.87	0.216
G2 (n = 10)	0 h	137.2 ± 36.78	11.97	0.003*	0h - 1h	-108.1	-4.05	0.001*
	1 h	245.3 ± 78.46			0h - 24h	-62.9	-2.35	0.005*
	24 h	200.1 ± 56.59			1h - 24h	45.2	1.69	0.073

The within-group comparison of TNF-α levels in GCF for G1 and G2 retraction cordless groups showed significant temporal changes. In G1, TNF-α levels increased significantly from baseline (0 h: 186.6 ± 84.01 pg/mL) to 1 h (277.5 ± 95.9 pg/mL; p = 0.002) and remained elevated at 24 h (250 ± 113.54 pg/mL; p = 0.004), indicating sustained inflammation (p = 0.001). In G2, TNF-α levels rose significantly from baseline (191.2 ± 74.29 pg/mL) to 1 h (248.8 ± 92.27 pg/mL; p = 0.001), but decreased significantly by 24 h (206.7 ± 91.4 pg/mL; p = 0.003), suggesting a transient inflammatory peak (p = 0.001). Kaolin-based paste induced a more persistent inflammatory response than the polyvinyl acetate-based strip, which showed a significant decline after 24 h (Table 5).

**Table 5:** Intragroup comparison of mean tumor necrosis factor-alpha (TNF-α) levels in gingival crevicular fluid (GCF) in picograms/mL (pg/mL) at multiple time intervals. *p < 0.05 denotes statistical significance using repeated analysis of variance with Bonferroni post-hoc test, G1 represent group using kaolin-based cordless retraction material, G2 represents group using polyvinyl acetate-based cordless retraction material. Data are presented as mean and standard deviation (SD), where n denotes number of patients in each group as a split-mouth study.

Groups	Time	Mean ± SD	F value	p-value	Repeated measure	Mean difference	t value	p-value
G1 (n = 10)	0 h	186.6 ± 84.01	19.25	0.001*	0h - 1h	-90.9	-4.06	0.002*
	1 h	277.5 ± 95.90			0h - 24h	-63.4	-1.44	0.004*
	24 h	250.0 ± 113.54			1h - 24h	27.5	0.62	0.181
G2 (n = 10)	0 h	191.2 ± 74.29	14.36	0.001*	0h - 1h	-57.6	-4.49	0.001*
	1 h	248.8 ± 92.27			0h - 24h	-15.5	-1.04	0.131
	24 h	206.7 ± 91.40			1h - 24h	42.1	3.09	0.003

## Discussion

The study revealed that both cordless gingival retraction systems induced a significant increase in IL-1β and TNF-α levels in the GCF at 1 h post-retraction, indicating an acute inflammatory response triggered by the mechanical and chemical interactions of these materials with gingival tissues. Merocel’s soft, expansive polyvinyl acetate structure induces prolonged IL-1β elevation through sustained mechanical pressure but allows quicker TNF-α resolution due to its biocompatibility and minimal chemical irritation [5]. Traxodent’s kaolin-based, abrasive, and astringent nature causes a rapid, transient IL-1β spike due to acute trauma but sustains TNF-α due to prolonged irritation or mediator retention [6]. The oral microbiome could also modulate cytokine responses. Traxodent’s astringent properties might disrupt microbial biofilms, releasing lipopolysaccharides (LPS) that sustain TNF-α production. Merocel’s inert nature may have less impact on microbial communities, contributing to quicker TNF-α resolution but prolonged IL-1β due to mechanical effects.

The immediate elevation in inflammatory biomarker levels aligns with the expected tissue response to the introduction of foreign materials into the gingival sulcus. The placement of retraction materials, whether kaolin- or polyvinyl-based, causes localized trauma and irritation, stimulating the release of pro-inflammatory cytokines, such as IL-1β and TNF-α, which are critical mediators of immune responses in periodontal tissues [7]. These cytokines play pivotal roles in initiating and amplifying inflammation, facilitating tissue remodeling, and signaling immune cell recruitment [10].

For the polyvinyl acetate-based strip, IL-1β levels remained significantly elevated at 24 h, suggesting a more sustained inflammatory response than that of the kaolin-based paste, where IL-1β levels showed a non-significant decline after 24 h. This finding indicates that the polyvinyl acetate-based material may provoke a prolonged inflammatory state, potentially owing to its physical properties, such as its strip-based structure, which may cause greater mechanical irritation or prolonged contact with the gingival tissues. In contrast, the kaolin-based paste exhibited a more transient IL-1β peak, suggesting that the kaolin-based material may be less irritating over time, possibly because of its injectable paste form, which allows for easier application and removal, minimizing sustained tissue trauma. Hong et al. conducted a systematic review to compare the efficacy of retraction paste and retraction cord in fixed prosthodontics, including nine studies [12]. They concluded that gingival retraction paste had a better effect on gingival health than the gingival retraction cord. Similar findings were reported by Wang et al. [13].

Conversely, for TNF-α, the kaolin-based paste showed a more persistent inflammatory response, with elevated levels at 24 h, whereas the polyvinyl acetate-based strip demonstrated a significant decline at the same time point. This differential response suggests that the chemical composition of kaolin-based paste, which includes kaolin and other hemostatic agents, may contribute to the prolonged cytokine release. Kaolin, a clay-based material, is known to provide hemostasis and tissue retraction through physical and chemical mechanisms, which might sustain inflammatory signaling compared with the more inert polyvinyl acetate used in polyvinyl acetate-based hemostatic strips [14]. It has been documented that kaolin facilitates coagulation by stimulating Factor XII, thereby initiating the intrinsic coagulation pathway through the activation of Factor XI, culminating in the production of a fibrin clot. Furthermore, kaolin can enhance the activation of platelet-associated Factor XI to initiate the intrinsic coagulation cascade, typically in individuals deficient in Factor XII [15].

These contrasting patterns in IL-1β and TNF-α responses highlight the complex interplay between material properties and biological responses, underscoring the need for careful material selection based on the clinical context. The observed increase in IL-1β and TNF-α levels at 1 h for both materials is consistent with previous studies on gingival retraction [10,16]. For instance, studies evaluating conventional retraction cords have reported similar elevations in GCF cytokine levels post-retraction, attributed to mechanical displacement and tissue trauma [10,16]. The acute inflammatory peak at 1 h reflects the immediate response to sulcular manipulation, a finding supported by Mathew et al., who noted increased TNF-α levels following cord-based retraction [10]. The transient nature of the IL-1β response in kaolin-based paste aligns with studies suggesting that cordless systems, owing to their less invasive application, cause reduced tissue trauma compared to cords [12,13].

The sustained IL-1β elevation in the polyvinyl acetate-based strip group could be attributed to the strip-based application, which requires packing into the sulcus, potentially causing prolonged mechanical stress. This is supported by research indicating that physical manipulation of gingival tissues, even with biocompatible materials, can exacerbate inflammation if the contact is prolonged [12,13]. In contrast, persistent TNF-α elevation with kaolin-based paste may be linked to its chemical composition. Kaolin-based materials often contain aluminum chloride or other astringents that can induce localized tissue irritation and are effective for hemostasis, as reported in studies comparing chemical retraction agents [14]. This chemical irritation may sustain TNF-α release, as TNF-α is particularly sensitive to chemical stimuli in periodontal tissues. Einarsdottir et al. reported more gingival recession with conventional retraction cords when used for impression-making than with aluminum chloride paste [17].

These findings partially align with those of Martins et al.’s systematic review, which concluded that conventional cord systems achieve greater gingival displacement but are associated with increased tissue trauma compared to cordless systems [7]. While their review focused on displacement efficacy, the current study extended the evaluation of inflammatory biomarkers and provided a biological perspective. The reduced IL-1β persistence with kaolin-based paste supports the notion that cordless systems are less traumatic; however, the sustained TNF-α response suggests that material-specific effects should be considered.

In contrast, Huang et al. compared the efficacy of cord and cordless retraction systems and found that out of seven included studies, three found better gingival health with a cordless or paste system, one reported increased gingival displacement with a paste system, and two reported no statistical difference in gingival displacement between the two systems [18]. The split-mouth design of the current study controlled for interindividual variability, thus strengthening the attribution of differences in the material properties. Madaan et al. compared the efficacy of different cordless gingival retraction systems and found that kaolin-based paste led to the least gingival displacement, and polyvinyl acetate-based strip led to the highest gingival displacement, which was consistent with previous studies [19,20]. However, the lack of significant intergroup differences in TNF-α levels at 24 h contrasts with the expectation that cordless systems universally reduce inflammation, indicating that material-specific effects may be cytokine dependent [21].

Clinical implications

These findings have significant implications for clinical restorative dentistry. Both cordless gingival retraction systems were effective; however, their differential inflammatory profiles suggest tailored applications. Kaolin-based paste may be preferred in patients with healthy gingiva or in those requiring rapid recovery, given its transient IL-1β response. However, sustained TNF-α elevation warrants caution in patients with compromised periodontal health because prolonged inflammation can exacerbate tissue damage. A polyvinyl acetate-based strip, with its sustained IL-1β response, may be better suited for cases where prolonged retraction is needed; however, clinicians should monitor for extended inflammation, particularly in patients prone to gingival sensitivity.

As demonstrated in this study, GCF biomarker analysis is a practical tool for assessing the biocompatibility of retraction materials. Measuring IL-1β and TNF-α levels can guide material selection and inform post-procedure care, such as recommending anti-inflammatory rinses or follow-up visits to monitor gingival health. Clinicians should also consider patient-specific factors, such as oral hygiene status and systemic health, when choosing retraction systems to minimize their adverse effects.

Limitations

Despite its robust design, this study had several limitations. First, the small sample size (n = 10) limited the generalizability of the findings. Although the split-mouth design and statistical power calculations ensured adequate sensitivity, larger studies are required to confirm these results across diverse populations. Second, this study focused solely on IL-1β and TNF-α, excluding other relevant biomarkers that could provide a more comprehensive picture of the inflammatory response. Third, the 24-h follow-up period may not capture long-term inflammatory effects, as some studies suggest that gingival recovery may take a longer time. Fourth, this study did not assess clinical outcomes, such as impression quality or patient comfort, which are critical for evaluating the practical utility of these materials. Finally, operator variability, despite calibration, could introduce subtle differences in material application, potentially influencing outcomes.

Future studies should address these limitations by incorporating larger sample sizes, additional biomarkers, and extended follow-up periods. Comparative studies evaluating cordless systems against conventional cords in terms of biological and clinical outcomes would further clarify their advantages. Additionally, investigating patient-reported outcomes, such as pain or discomfort, could enhance the clinical relevance of these findings.

## Conclusions

This study demonstrated that both cordless gingival retraction systems induced an acute inflammatory response in the gingival tissues, as evidenced by the elevated levels of IL-1β and TNF-α in the GCF. The kaolin-based system (Traxodent®) exhibited a more transient IL-1β response; however, it sustained TNF-α elevation, while the polyvinyl acetate-based system (Merocel®) showed prolonged IL-1β elevation with a quicker resolution of TNF-α. These distinct inflammatory profiles highlight the influence of material composition on periodontal tissue responses. These findings emphasize the importance of selecting retraction materials based on patient-specific factors and clinical requirements to minimize gingival irritation while ensuring effective retraction. Monitoring inflammatory biomarkers in GCF offers a valuable approach to assess the biocompatibility of retraction systems, guiding clinicians to optimize restorative procedures while preserving periodontal health.
